# Concentrations of Soluble Angiotensin Converting Enzyme 2 (sACE2) in Children and Adults with and without COVID-19

**DOI:** 10.3390/jcm11226799

**Published:** 2022-11-17

**Authors:** Sarah Isabella Wissing, Rima Obeid, Tanja Rädle-Hurst, Tilman Rohrer, Christian Herr, Jakob Schöpe, Jürgen Geisel, Robert Bals, Hashim Abdul-Khaliq

**Affiliations:** 1 Department of Pediatric Cardiology, Saarland University Hospital, 66421 Homburg, Germany; 2 Department of Clinical Chemistry and Laboratory Medicine, Saarland University Hospital, 66421 Homburg, Germany; 3 Department of Pediatric Endocrinology, Saarland University Hospital, 66421 Homburg, Germany; 4 Department of Internal Medicine V–Pulmonology, Allergology and Critical Care Medicine, Saarland University Hospital, 66421 Homburg, Germany; 5 Institute for Medical Biometry, Epidemiology and Medical Informatics, Saarland University Medical Center, 66421 Homburg, Germany; 6 Helmholtz Institute for Pharmaceutical Research Saarland (HIPS), Helmholtz Centre for Infection Research (HZI), Saarland University Campus, 66123 Saarbrücken, Germany

**Keywords:** soluble ACE2, COVID-19, pediatrics, age dependence, severity of COVID-19, adults with COVID-19, children with COVID-19

## Abstract

Severe acute respiratory syndrome coronavirus 2 (SARS-CoV-2), causing the coronavirus disease 2019 (COVID-19) pandemic, leads to illness and death. Various risk factors for a severe course, such as higher age, male gender and pre-existing illnesses are known. However, pathophysiological risk factors are largely unclear. Notably, the mild course of disease in children is conspicuous. Angiotensin converting enzyme 2 (ACE2) serves as a receptor for SARS-CoV-2 and is a key enzyme in infection. Differences in the distribution of ACE2 can provide insights into different courses of COVID-19. Our aim was to elucidate the role of ACE2 as a pathophysiological risk factor by measuring soluble ACE2 (sACE2) via ELISA in blood samples (lithium-heparin-plasma or serum) of 367 individuals including children and adults with and without COVID-19. sACE2-levels were compared between the groups according to age and sex. In adults and children with COVID-19, sACE2-concentrations are significantly higher compared to healthy individuals. sACE2-levels increase with age and are lower in children compared to adults with COVID-19. Sex doesn’t significantly influence sACE2-concentration. It remains unclear whether sACE2 concentrations increase because of the infection and what factors could influence this response. In conclusion, the increase of sACE2-concentration with age could indicate that ACE2 concentrations mirror increased COVID-19 severity.

## 1. Introduction

The coronavirus disease 2019 (COVID-19) pandemic emerged in December 2019 due to the severe acute respiratory syndrome coronavirus 2 (SARS-CoV-2) and is responsible for illness and cases of death all over the world. While various risk factors for a severe course of the disease, such as higher age, male gender, obesity and pre-existing illnesses are known [1,2], it is largely unclear which pathophysiological differences cause the increased risk. Notably, the mild course of the disease in children is conspicuous [3].

Key enzymes for infection with SARS-CoV-2 is the angiotensin converting enzyme 2 (ACE2). It is a membrane bound carboxypeptidase that is expressed on the surface of cells in many organs, such as the kidney, heart, gastro-intestinal-tract and the lung [4]. ACE2 converts different peptides. Important is the counterregulatory function of ACE2 to ACE in the renin-angiotensin-aldosterone-system (RAAS) [5]. While ACE converts angiotensin I (AT1) to angiotensin II (AT2), which has a vasoconstrictive effect, ACE2 converts AT1 to Angiotensin-1-9, and AT2 to angiotensin 1-7, which have vasodilative and anti-inflammatory functions [5,6]. Via shedding of ACE2 from the membrane by the adisintegrin-like and metalloproteinase 17 (ADAM17), soluble ACE2 (sACE2), which circulates in the blood, is formed [5].

We have to differentiate between soluble sACE2 and membrane bound mACE2. The mACE2 is mainly responsible for the cell entry of SARS-CoV-2 [7]. mACE2 interacts with the spike protein, which is located on the surface of the virus [8]. After priming the spike protein with the transmembrane protease serine subtype 2, the virus binds to ACE2 and initiates the fusion to the host cell [9]. However, the role of sACE2 in the COVID-19 infection is unclear. Data from an in vitro study showed that the cell entry of SARS-CoV-2 is also possible via sACE2. After binding to sACE2, the entry to the host cell occurs via endocytosis [10]. Differences in the distribution of ACE2 can give insights into different courses of COVID-19.

For several diseases, mainly cardiovascular, it is known that there is an association to high sACE2-concentration, such as hypertension, diabetes, obesity or renal disease [11,12,13,14], which are also known as risk factors for severe COVID-19 [2,15]. Therefore, high sACE2 could be a risk factor for severe COVID-19. We have demonstrated that sACE2 concentration in serum was associated with the severity of congenital heart diseases and heart failure in adults [16].

During the pandemic, children and young adults usually showed a mild courses of COVID-19 [3]. Therefore it is interesting to elucidate the pathophysiological mechanism and the role of sACE2 in children in comparison to adults. The identification of pathophysiological risk factors is relevant for a deeper understanding of the infection process, for the protection of risk groups and for the development of therapeutic approaches to COVID-19. In this study, we investigated the concentration of sACE2 in the plasma of children, as ACE2 is an important factor for infection with SARS-CoV-2. To date there is little data available on sACE2 concentrations in children [17,18], and as far as we know, data on sACE2 in children with COVID-19 is missing.

## 2. Materials and Methods

### 2.1. Collection of the Samples

The study was performed at the University-Hospital of Saarland, Germany. Blood-samples of the following four groups were used for assessment of sACE2 concentrations: children and adults with COVID-19, pediatric patients without COVID-19, and healthy individuals who served as controls.

The samples of patients with COVID-19 were collected as part of the “CORSAAR-” or “CORSAAR-Pädiatrie”-study. The aim of both studies is to evaluate risk factors for SARS-CoV-2 infection, the mechanisms of COVID-19-infection, and the identification of new therapy options [19]. All subjects gave informed and written consent to participate in the study. The patients tested positive for SARS-CoV-2 via real-time PCR and were treated in an inpatient setting. No child needed mechanical ventilation and no child died. The serum samples were centrifuged for 20 min at 2500× *g* and 4 °C, aliquoted and stored below −70 °C. The samples we used were collected between April and December 2020. The exact protocol of the study has already been published [20].

We used the residua of blood-samples taken for routine laboratory studies of pediatric patients. The lithium-heparinat samples were collected between June and September 2020. Samples were collected due to a clinical indication and centrifuged at 4000 rpm for 10 min. After the clinically relevant laboratory tests were completed, the remaining plasma samples were stored at 4 °C overnight and made anonymous. The samples used for our research work were randomly selected from all anonymized samples. One day after samples were taken from the patient, 300–500 μL of plasma was aliquoted, which was immediately frozen and stored at −20 °C.

Healthy individuals served as controls. The serum samples were collected as part of a study on adults with congenital heart disease performed in our institution. Healthy volunteers answered a questionnaire, and cardiac echo and blood examinations were used to prove their health conditions. The study protocol has already been described in detail and the Saarland Medical Association ethical board approved the study (no. 73/09) [16,21]. The samples were collected between January 2015 and December 2019, and all subjects gave written and informed consent.

To verify that there is no difference in sACE2-concentration in serum and lithium-heparinat-plasma samples, we collected samples of both materials at the same time from the same patient during cardiac catheter examination or when a blood sample was needed due to a clinical question. The patient or legal guardian gave the informed and written consent.

The ethics committee of the Saarland medical association or the ethics committee of the Saarland University Hospital approved the study.

Hemolytic samples or those with a dark color were excluded from the study. As we wanted to avoid samples of the same patient and the samples were anonymized, we sorted them by the date of birth and sex. Of samples with the same parameters in both categories, only the first one was used for evaluation in our study.

### 2.2. Biochemical Analysis

A commercially available sandwich enzyme-linked immunosorbent assay (ELISA) (Human ACE2^®^, Cloud-Clone Corporation, Houston, TX, USA) was used to measure the sACE2 concentration in the blood samples. The measurement was performed according to the instruction manual. We used a 10-fold dilution of the samples prior to measurement.

The samples were measured on a FLUOstar^®^ Omega Microplate Reader (BMG LABTECH Inc., Cary, NC, USA).

### 2.3. Statistical Analysis

For our statistical analysis we used SPSS (SPSS Version 25; SPSS Inc., Chicago, IL, USA).

As the variables are not normally distributed, they are given as median and interquartile range (IQR). The IQR represents the value of the 25th and 75th percentile. In diagrams, the 95% confidence interval and the median are shown. A level of 2 pg/mL was given to all samples with sACE2 concentrations that were below the lowest calibrator of the assay.

For descriptive statistics, the median (IQR) and the distribution of the variables age, gender and sACE2 in the different groups and all measured values were determined.

A Mann-Whitney-U-test was used to investigate differences between groups. To verify that sACE2-concentrations are similar in serum and plasma we used a Wilcoxon-test. To study the association between age, sex and concentrations of sACE2 we used a linear multivariate regression analysis within the four different groups. 

We considered a *p*-value < 0.05 as statistically significant.

## 3. Results

### 3.1. Descriptive Statistics

A total of 367 individuals were included in the study. Regarding all pediatric and adult individuals, the median age was 10.75 (2.08–18.17) years, and 42.7% (152/356) were female. Female individuals were 10.21 (2.63–18.57) and male individuals were 10.92 (1.17–18.78) years old. The characteristics of the different groups are shown in the table below (Table 1).

To verify that there was no difference in sACE2 in serum and plasma samples we compared the sACE2-concentration in serum and plasma samples in 12 individuals (5 male, 7 female) with an age of 4.5 (2–17.25) years. We did not find a significant difference using a Wilcoxon-test (*p* = 0.110).

### 3.2. sACE2 and Sex

**Figure 1:**
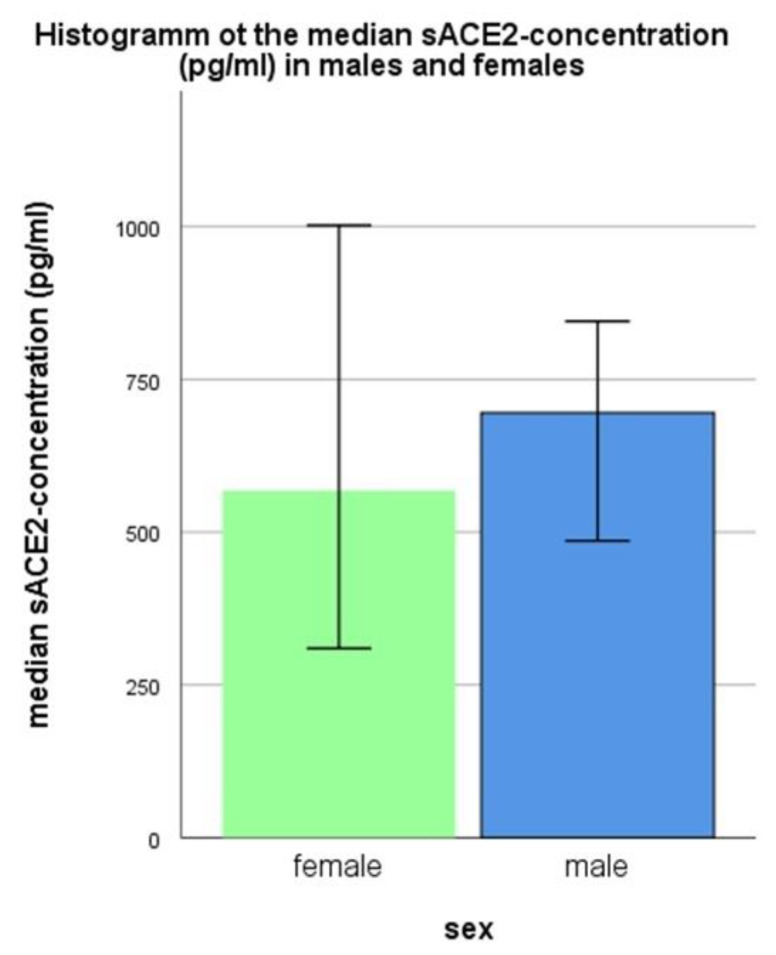
Association of sex and sACE2-concentration within all individuals included in the study (median and 95% confidence interval is shown).

Regarding the sex of all individuals included in the study, the median sACE2-concentration in females is slightly lower than in males, (median 567.73 pg/mL (141.75–1772.49) vs. 695.52 pg/mL (168.36–1927.56)) (Figure 1). However, this difference reaches no statistical significance including all test persons (*p* = 0.441), or within any of the groups (pediatrics *p* = 0.618, adults with COVID-19 *p* = 0.601, children with COVID-19 *p* = 0.919, healthy controls *p* = 0.174).

### 3.3. sACE2 and Age

**Figure 2:**
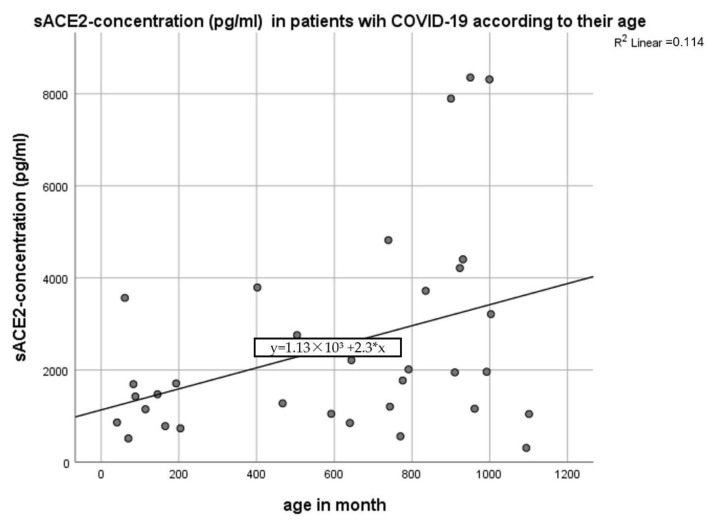
Correlation of sACE2 concentration (pg/mL) and raising age (in month) in patients with COVID-19, linear multivariate regression analysis (age and sex), p = 0.03.

Interestingly, the sACE2-concentration increases significantly with the age in patients with COVID-19 (children and adults) (B = 2.309 ± 1.012, *p* = 0.03) (Figure 2).

In using a linear regression analysis for age and sex, there is no significant correlation of age and sACE2-level in any other group (pediatrics (*p* = 0.184) adults with COVID-19 (*p* = 0.364), children with COVID-19 (*p* = 0.510) and adult healthy controls (*p* = 0.558)). Spliting all included individuals in subgroups under and above 18 years of age and comparing them using a Mann-Whitney-U-test, we could not find a significant correlation between age and the level of the sACE2, either (*p* = 0.94).

### 3.4. Comparison of sACE2 in Different Groups

**Figure 3:**
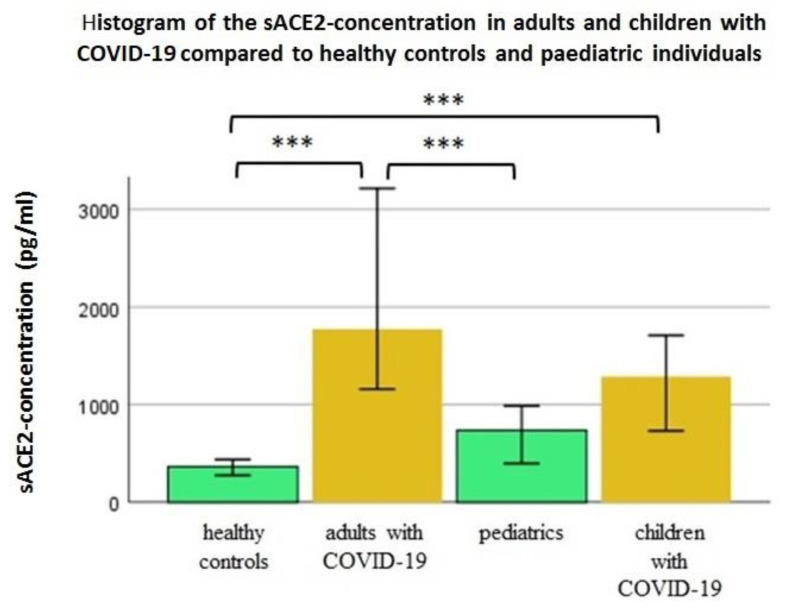
Comparison of the sACE2-cocentration in different age-groups with and without COVID-19, Mann-Whitney-U-test, *** significant difference: *p* < 0.001 (median and 95% confidence interval shown).

Adults and children with COVID-19 have significantly higher sACE2-levels than healthy individuals (*p* < 0.001). Compared to the pediatric patients, adult patients with COVID-19 have significantly higher sACE2-concentraitons than children (*p* < 0.001). For the children with COVID-19, the median sACE2-concentration is higher in healthy children, but this difference is not significant (*p* = 0.222). Adult healthy controls and individuals from pediatrics do not differ significantly (*p* = 0.065) (Figure 3).

## 4. Discussion

According to several reports during the pandemic, there were significantly different patterns of disease manifestation of COVID-19 in children and adults. The course of COVID-19 in pediatric patients was mild and the number of hospitalizations and mortality was significantly lower. Severe disease in pediatric patients was reported in children in association with other severe diseases. [3,22,23].

Different explanations were discussed such as immunity, maturation of the immune system, less comorbidities as well as changes of the ACE2 receptors in the respiratory system [22,24].Thus, Bunyavanich et al. [25] found a significant increase of the ACE2 gene expression in the nasal epithelium with increasing age, suggesting higher predisposition for severe infection in adults than children. The relationship between the soluble circulating sACE2 and cell membrane bound mACE2, however, is not clearly understood [11,26]. Measurement of mACE2 in vivo is difficult because of the lack of biopsies in children and adults. Thus, the aim of this study was to analyze the serum level patters of ACE2 in adults and children with and without COVID-19 infection.

In this study, the sACE2-concentration in the blood was measured in children and adults with COVID-19 and compared to pediatric patients and adult healthy controls. Like other reports, we found higher sACE2 concentrations in association with COVID-19 infection in children and adults. While pediatric patients demonstrated higher concentrations of sACE2 than healthy adult controls, in patients with COVID-19 the sACE2-concentration increases significantly with age. 

### 4.1. Selection of the Test Person and Design of the Study

In contrast to other studies that measured the sACE2 concentration in the blood of COVID-19-patients, we worked with a healthy control group. For example, Rieder et al. [27] examined the sACE2 concentration of patients with respiratory symptoms with and without COVID-19, and Lerner et al. [28], who measured the sACE2 concentration in COVID-19 patients during the course of the disease, lacked a healthy control group. This is a strength of our study, as the use of comparison groups allows us to put our results in the context of the healthy general population.

To compare sACE2 values in children, a comparison group with healthy children would have been desirable. Since a blood sampling without medical indication in children is mostly ethically questionable, residual blood from medically indicated blood tests to exclude abnormal findings were used.

The use of a sandwich ELISA is common for sACE2 quantification and has been used in previous studies [27,28,29,30].

### 4.2. Higher sACE2 in Patients with COVID-19 and Possible Implications

We were able to demonstrate the different sACE2-levels in the blood of children and adults with and without COVID-19. Our study shows that pediatric and adult patients with COVID-19 have significantly higher sACE-2 levels than healthy controls. Higher levels of ACE2 in patients with COVID-19 were also found by Lundström et al. [31]. Moreover, Reindl-Schwaighofer et al. [32] also found an increased sACE2 concentration in patients with a severe course of COVID-19. Patel et al. [33] demonstrated higher sACE2 activity in recovered COVID-19 patients compared to healthy individuals. An association between sACE2 activity and sACE2 concentration was previously described by Zhang et al. [14].

Surprisingly, Osman et al. [34] reported a reduced sACE2 concentration in patients with prolonged virus excretion compared to healthy individuals. Moreover, Rieder et al. [27] described no differences in the sACE2 concentration between patients with respiratory symptoms related to COVID-19 and other non-COVID-19 respiratory diseases. This could be explained by the small cohort size used in these studies or the different times at which the samples were taken after the onset of the disease. Whether other respiratory diseases may also influence sACE2 is unclear. 

#### Age Dependency

We were able to demonstrate that the sACE2 level in adults with COVID-19 is significantly higher than the sACE2 level in pediatric patients and adult healthy persons. This result allows two possible hypotheses, which are explained in detail below: (1) high sACE2 could represent a risk factor for infection with SARS-CoV-2 and a severe course of the disease; and (2) infection with SARS-CoV-2 could increase the sACE2-level via the virus itself or the associated inflammation.

With regard to (1), our result, indicating that the sACE2-concentration is higher in ill persons, is consistent with the study by Rice et al. [13], which describes an association between various pre-existing illnesses (e.g., cardiovascular, diabetes, obesity) and a high sACE2 concentration. Treskova-Schwarzbach et al. [2] identified different pre-existing illnesses as a risk factor for a severe course of COVID-19. 

Taken together, this could indicate that a high sACE2 concentration is a risk factor for infection with SARS-CoV-2 or a severe course. Interestingly, Yeung et al. [10] showed in their in vitro experiments that higher sACE2 in the physiological range facilitated the infection of cells. Furthermore, high sACE2 values (>7200 pg/mL) in COVID-19 patients are, according to Lerner et al. [28], associated with the need for ventilation. This supports the hypothesis of high sACE2 levels being a risk factor. To test the hypothesis of sACE2 as a risk factor, a prospective study that examines the sACE2 level and the risk of developing COVID-19 could be worthwhile.

With regard to (2), the significant difference in the sACE2 level of healthy individuals to the individuals with COVID-19 suggests that SARS-CoV-2 itself or the pathophysiological changes associated with the infection, such as inflammation, increase sACE2 concentrations. Accordingly, Reindl-Schwaighofer et al. [32] describe higher sACE2-levels in patients with severe COVID-19. As it is known that SARS-CoV-1 sheds ACE2 from the cell [35,36], this could also be presumed for SARS-CoV-2. Hypoxia, a typical consequence of COVID-19, can also increase the sACE2 concentration [37]. Studies examining the effects of SARS-CoV-2 infection on the cellular level can provide further insights.

Interestingly, very high doses of recombinant human sACE2 can neutralize SARS-CoV-2 [38], which was successfully used in a case study [39]. Thus, depending on the concentration, sACE2 seems to play a dual role.

### 4.3. sACE2 in Children with and without COVID-19

We included children and adolescents in our measurements, which is a strength of our study. To date, few studies have been conducted on sACE2 in children [17,18] and to our knowledge this is the first time that sACE2 levels in children with COVID-19 were measured and compared to other groups. This is relevant because high age is a major risk factor for a severe course of COVID-19, and children usually have mild disease [1,3].

Different studies in healthy persons found an increase of sACE2 with age. Pavel et al. [17] found higher sACE2 in adults compared to children younger than five years, and Swärd et al. [18] found an increase in the sACE2 concentration during puberty. At the level of gene expression in nasal epithelial cells, Bunyanvanich et al. [25] also found lower gene expression of ACE2 in children. Our finding of a significant positive correlation between rising age and sACE2 matches with the studies described above.

Children with COVID-19, in our study, had lower sACE2 concentrations than adults with COVID-19. Even if this difference is not significant, it might be a clue to the special role of sACE2 in terms of a milder course in children compared to adults, since high sACE2 [32] and high age [1] are associated with a severe course.

### 4.4. sACE2 and Sex 

According to previous studies, sACE2 concentration was lower in females than in males. However, in our study, the difference was not significant. In other studies, significantly higher sACE2 values were found in males [17,31]. This is relevant, as males are at risk for a severe course of COVID-19 [40]. Our groups were not matched for disease severity according to sex, which may explain why we could not find a significant difference.

### 4.5. Limitations of the Study

Due to our study design, which used already anonymized residual samples, no clinical data such as medication or outcome of the test person were available in many patients. Some of these factors could influence the sACE2-concentration. Thus, the transferability of our results to the healthy general population could be limited. In further studies investigating the sACE2-concentration, it would be useful to know the clinical characteristics of the subjects to be able to quantify possible influencing factors, such as pre-existing illnesses, on the sACE2 concentration.

All patients with COVID-19 were treated as inpatients. Thus, the significance of our results for patients with a mild course is limited.

The cohort size of patients with COVID-19 (31 adults and 10 children) is quite small. This could skew the results. Therefore, further studies should be carried out with larger cohorts. However, most pediatric COVID-19 patients have mild symptoms and do not require clinic admission. 

In this study, we chose blood samples as test material because they are quite easy to obtain and they also provide an insight into the importance of sACE2 in vivo. However, it should be noted that the sACE2 concentration in the blood does not necessarily correlate with the membrane-bound form [11,26]. In addition, the first contact with SARS-CoV-2 usually occurs via the mucous membranes of the respiratory tract [41], and the virus spreads within the body and via the blood afterwards. Thus, other protective mechanisms can also be important during the initial infection, about which no statement can be made from the selected sample material.

### 4.6. Perspective

COVID-19 will be of importance into the future. Therefore, it is relevant to understand the pathophysiology of the disease as best as possible. Our study has provided new insights into the importance of sACE2 in patients (and especially children) with COVID-19. Our data suggest that higher blood sACE2 levels could predispose individuals to COVID-19 and a severe course of the disease. Based on these findings, further research work can be set up, by measuring the sACE2 concentration in the blood of large cohorts with clinically well-characterized subjects and control groups, using healthy children and adults. Using a prospective study design and measuring the sACE2-concentrations before and during infection, relations between sACE2 concentration and the risk of contracting COVID-19 and suffering a severe course can be examined. Based on this, sACE2 can possibly be used as a risk marker in everyday clinical practice for patients with COVID-19.

## 5. Conclusions

In conclusion, we could find an association of high age, which is known as risk factor for a severe course of COVID-19, and high sACE2-concentration in patients with COVID-19. Moreover, sACE2-concentrations were significantly elevated in patients with COVID-19 compared to healthy adults. This work helps us to gain insights in the role of sACE2 in COVID-19 and provides new approaches in understanding the mild form of COVID-19 in children.

## Figures and Tables

**Table 1:** Overview of the number of test person, age, sex and sACE2 concentration (pg/mL) in the different groups, given as median (interquartile range (25–57 percentile)).

	Number of Test Persons	Age (at Enrolment) in Years Median (IQR (25.–75. Percentile))	Sex (Female/All)	Concentration of sACE2 (pg/mL) Median (IQR (25.–75. Percentile))
Pediatrics	263	5.25 (0.7–13.3)	113/260 (43.5%) 3 missing	704.463 (102.00–2017.798)
Adults with COVID-19	31	69.6 (53.7–80.1)	10/23 (45.5%) 8 missing	1772.49 (1043.1–3792.24)
Children with COVID-19	10	8.4 (5.6–14.3)	4/10 (40%)	1286.45 (768.34–1696.819)
Healthy controls (adults)	63	30 (22–38)	25/63 (39.7%)	365.2 (237.7–656.3)
Total	367	10.75 (2.08–18.17)	152/356 (42.7%)	695.52 (159.84–1838.39)

## Data Availability

Data is available upon request.
